# Origin of Limiting and Overlimiting Currents in Bipolar Membranes

**DOI:** 10.1021/acs.est.2c09410

**Published:** 2023-06-21

**Authors:** Ragne Pärnamäe, Michele Tedesco, Min-Chen Wu, Chia-Hung Hou, Hubertus V.M. Hamelers, Sohum K. Patel, Menachem Elimelech, P.M. Biesheuvel, Slawomir Porada

**Affiliations:** † Wetsus, European Centre of Excellence for Sustainable Water Technology, Leeuwarden, The Netherlands; ‡ Environmental Technology, Wageningen University, Wageningen, The Netherlands; § Graduate Institute of Environmental Engineering, National Taiwan University No.1, Sec. 4. Roosevelt Rd., Taipei 10617, Taiwan; ⊥ Department of Chemical and Environmental Engineering, Yale University, New Haven, Connecticut 06520-8286, United States; ∥ Department of Process Engineering and Technology of Polymeric and Carbon Materials, Wroclaw University of Science and Technology, Wyb. Wyspianskiego 27, Wroclaw 50-370, Poland

**Keywords:** bipolar membrane, water dissociation, water formation, limiting current, pH control, chemical production, ionotronics

## Abstract

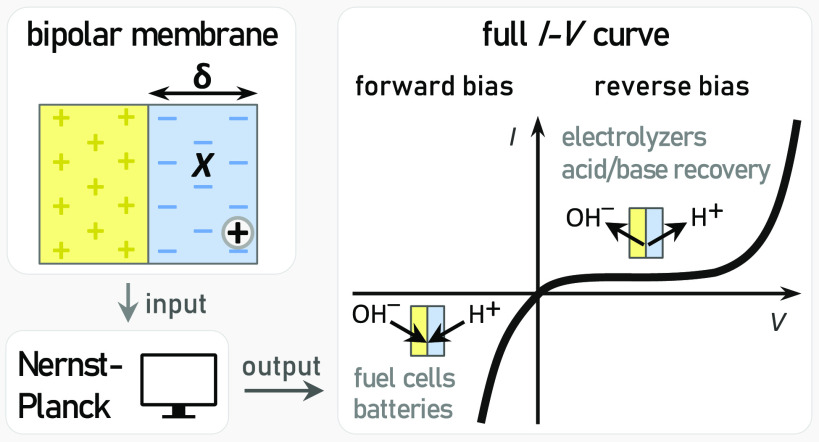

Bipolar membranes (BPMs), a special class of ion exchange membranes with the unique ability to electrochemically induce either water dissociation or recombination, are of growing interest for environmental applications including eliminating chemical dosage for pH adjustment, resource recovery, valorization of brines, and carbon capture. However, ion transport within BPMs, and particularly at its junction, has remained poorly understood. This work aims to theoretically and experimentally investigate ion transport in BPMs under both reverse and forward bias operation modes, taking into account the production or recombination of H^+^ and OH^–^, as well as the transport of salt ions (e.g., Na^+^, Cl^–^) inside the membrane. We adopt a model based on the Nernst–Planck theory, that requires only three input parameters—membrane thickness, its charge density, and p*K* of proton adsorption—to predict the concentration profiles of four ions (H^+^, OH^–^, Na^+^, and Cl^–^) inside the membrane and the resulting current–voltage curve. The model can predict most of the experimental results measured with a commercial BPM, including the observation of limiting and overlimiting currents, which emerge due to particular concentration profiles that develop inside the BPM. This work provides new insights into the physical phenomena in BPMs and helps identify optimal operating conditions for future environmental applications.

## Abstract 2

Development and application of bipolar membranes is thriving. We present a trackable model to predict the behavior of a commercial bipolar membrane throughout its entire operational window (water dissociation and recombination).

## Introduction

Bipolar membranes (BPMs) represent a distinct class of ion exchange membranes with the unique ability to electrochemically dissociate water into protons and hydroxide ions.^1^ These membranes have become increasingly attractive for the production of acid/base and for emerging environmental applications which aim to phase out the dosing of harsh chemicals for pH regulation^2−8^ as well as other applications at the water-energy nexus.^9−13^ Specifically, BPMs are ion exchange membranes with two oppositely charged layers: an anion exchange layer (AEL) and a cation exchange layer (CEL). Due to such a composite structure, the function of a BPM is not selective ion separation as neither anions nor cations can ideally pass both layers of the membrane. Instead, a BPM is used for its ability to regulate and maintain a different pH on either side via a water dissociation reaction at the BPM junction.^14−17^

**Figure 1:**
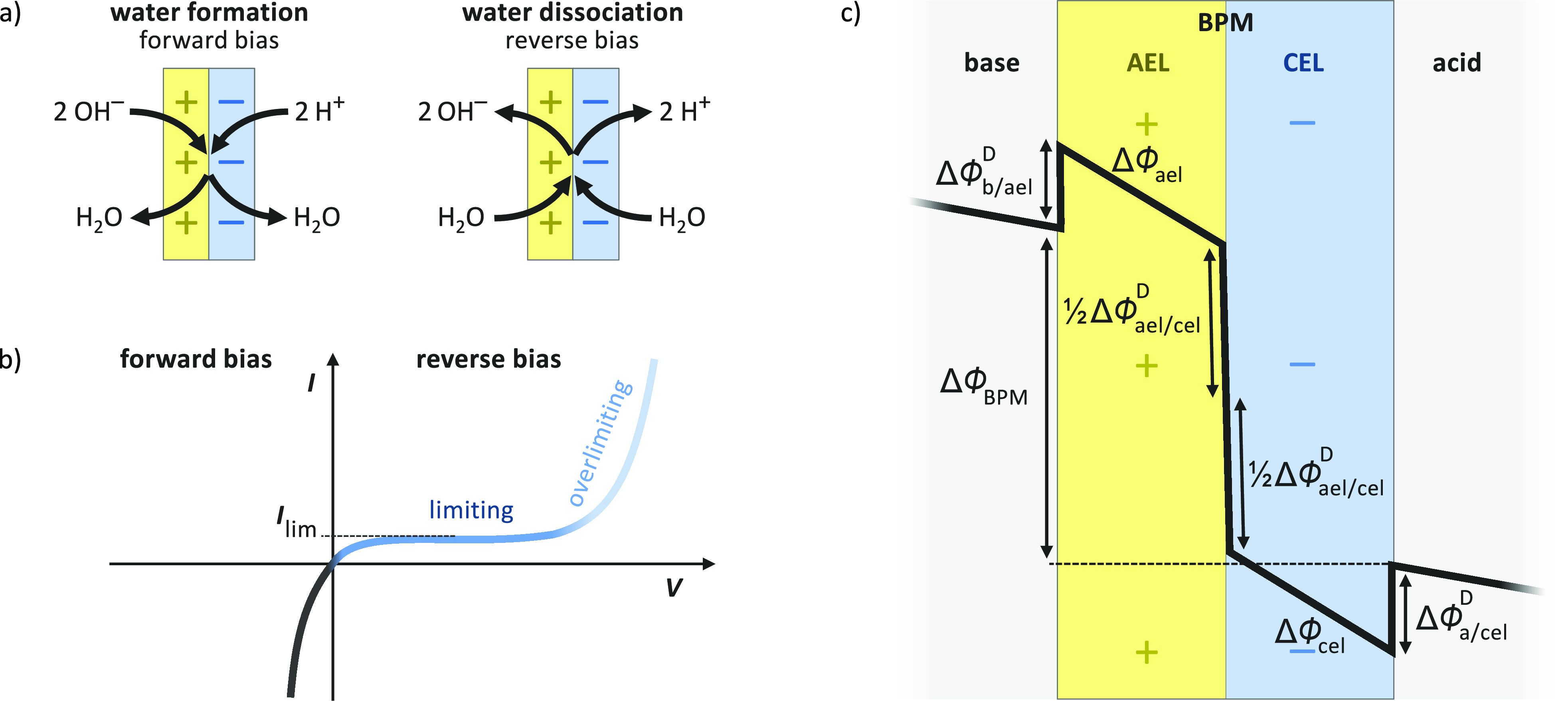
(a) Operational modes of a bipolar membrane (BPM). Under forward bias water is formed in the BPM junction by recombining H^+^ and OH^–^ from the opposite sides of the membrane. Under reverse bias water is dissociated into H^+^ and OH^–^ ions, creating an acidic and a basic solution on opposite sides of the membrane. )b) Typical current–voltage (*I*–*V*) curve of a BPM in neutral conditions. At voltages below the open circuit potential the membrane is under forward bias. At voltages above the open circuit potential the membrane is under reverse bias, which can be divided into a limiting current region and an overlimiting current region. (c) Schematic representation of the electric potential profile across a BPM. The BPM voltage is a sum of potential change through two ion exchange layers and Donnan potential differences at three interfaces (two between the membrane and electrolyte and one between the membrane layers).

Because a BPM consists of oppositely charged layers, the orientation of a BPM in an electric field is important. The BPM must be facing the cathode with its CEL to facilitate water dissociation (Figure 1a). In such an orientation, the BPM is under reverse bias, where the membrane gets depleted of ions. To withstand the current, water is dissociated into H^+^ and OH^–^ ions at the BPM junction. The produced “water ions” can leave the BPM only through the respective BPM layer, thus creating an acidic and basic solution on opposite sides of the membrane. Accordingly, BPMs are nowadays mostly used for the production and recovery of inorganic acids and bases from salt solutions (e.g., from NaCl, NaNO_3_, Na_2_SO_4_, and NH_4_F^1,18−21^). However, various other applications have been proposed, including CO_2_ capture,^6,22^ lithium recovery,^3^ production of alkoxides (i.e., by alcohol dissociation at the BPM junction^23^), recovery of ammonia from wastewater,^8^ and more recently, BPM assisted electrolyzers,^11,24^ fuel cells,^9,12^ electrosorption,^25^ and batteries.^13,26−28^ Notably, the latter three applications involve the use of BPMs not only under reverse but also forward bias (CEL facing the anode), to recombine H^+^ and OH^–^ ions to water (Figure 1a). An acid–base flow battery (ABFB), for example, operates with alternating bias. During charging (reverse bias), an external current is applied to the ABFB to dissociate water at the BPM junction and generate acid and base from an aqueous salt feed solution, thus storing electricity in the form of chemical energy. During discharge (forward bias), the process is reversed: H^+^ and OH^–^ ions from acid and base are recombined into water at the BPM junction, and the ionic current generated in the battery stack by salt ions is harvested as electrical current at the electrodes.^13^

With the exception of the BPM assisted fuel cell (which uses H_2_ and O_2_ gases as feed^12^), none of the applications mentioned above contain only H^+^ and OH^–^ but also other ions (e.g., Na^+^ and Cl^–^ in the example of the ABFB). Due to the nonideal selectivity of practical BPMs, these “salt” ions cross the BPM to some extent,^29^ leading to salt ion crossover (also called ”salt leakage”) and decreased current efficiency in the system. Therefore, when studying transport phenomena in BPMs and at membrane/solution interfaces, it is important to consider the transport of all species in the solution.

In this work, we present a theoretical framework to explain the current–voltage (*I*–*V*) behavior of a BPM in neutral (aqueous NaCl solution) but also in extreme pH (0.5 M HCl and 0.5 M NaOH) conditions, over a wide operational window. We focus on both operating modes (Figure 1a)—forward bias (water formation) and reverse bias (water dissociation)—and take into account the transport of both water ions (H^+^, OH^–^) and salt ions (Na^+^, Cl^–^) through the membrane. We consider two cases in particular: (i) the same neutral pH salt solution on both sides of the BPM, and (ii) an acid and a base solution on opposite sides of the BPM. To investigate the competitive transport effects between H^+^ and OH^–^ ions and salt ions, in case ii, we also test acid and base solutions that have varying (but equal for both solutions) concentration of salt added as background electrolyte. We validate our model by comparing the *I*–*V* curves predicted by the model to *I*–*V* curves measured with a commercial BPM. To the best of our knowledge, this work is the first to predict BPMs’ behavior over such a wide operating window, including under both biases. This makes the hereby described model especially interesting for applications that use BPMs under forward bias, such as batteries, electrosorption, and fuel cells. Furthermore, the simplified modeling framework presented here may be extended upon for the development of emerging BPM technologies applied to more complex environmental systems.

## Theory of Ion Transport in Bipolar Membranes

The theory presented in this work is based on the Nernst–Planck approach for describing ion transport in ion exchange membranes,^30,31^ and it is made with the presumption of perfect symmetry for simplification. Thus, we consider a symmetric BPM and assume that the AEL and CEL have equal thickness, the same transport properties for respective co- and counterions, and equal fixed charge density, except for the opposite sign. In addition, we consider NaCl as the only 1:1 fully dissociated electrolyte in the solution, and assume equal diffusion coefficients for the two salt ions  and for the two water ions , with water ions having 5 times higher diffusion coefficient than the salt ions (i.e., in calculations of this work, we take a factor of 5 difference between the water ions and the salt ions). In case of the BPM submerged in electrolyte solution, we assume equal salt concentration on either side of the BPM; in case of the BPM separating an acid and base solution, we assume that the concentration of acid on one side equals to the concentration of base on the other side. While the latter case might seem asymmetric, it is symmetric from a theoretical perspective, with AEL and CEL in similar conditions (except for different signs and directions of fluxes). Due to the assumption of symmetry, the junction always has a pH of 7 and equal concentrations of Cl^–^ and Na^+^ ions. We presume that in the AEL the concentration of H^+^-ions can be neglected, while in the CEL the OH^–^-ions can be neglected. Thus, in each ion exchange layer, three ions are considered (i.e., in the AEL: OH^–^, Cl^–^, Na^+^; in the CEL: H^+^, Na^+^, Cl^–^). The molar flux of each ion, *J*_*i*_, is described by the Nernst–Planck equation, which describes ions as ideal point charges
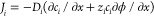
1where *x* is a directional coordinate across the BPM, *c*_*i*_ the ion concentration inside the BPM, *z*_*i*_ the valence of the ion, ϕ the dimensionless electric potential, and *D*_*i*_ the ion diffusion coefficient in the membrane, generally ∼10–100 lower than in free solution.^32^ Under steady-state conditions, this flux is invariant across the ion exchange layers (IELs). At each position in the two layers (both layers with a thickness δ_iel,*i*_) we have local electroneutrality, *∑*_*i*_*z*_*i*_*c*_*i*_ + *ωX* = 0, where *X* is the membrane charge density and ω the sign of the membrane charge, which is ω = −1 in the CEL, and ω = +1 in the AEL. The ionic current density through the AEL and CEL, *I*, is a summation over all ions, *I* = *F ∑*_*i*_*z*_*i*_*J*_*i*_, and is the same at each *x*-position in the BPM. Because we assume steady state conditions, there is no accumulation of ions in the membrane: the undesired co-ion fluxes of Cl^–^ and Na^+^ ions across the CEL and AEL, respectively, continue unchanged throughout the entirety of the BPM (i.e., the flux of each of the salt ion across the AEL and CEL is equal). Due to the perfect symmetry assumption, the fluxes of the salt ions are also equal in magnitude (but opposite in direction). Thus, the flux of the counterions in a certain IEL, *J*_ct_, is the same as the flux of co-ions there, *J*_co_, only with an opposite sign: . The voltage across the BPM, *V*_bpm_, is a sum of the potential change through both IELs and the Donnan potential differences across all three interfaces (Figure 1c). Because of symmetry of the calculations, we can take only half of the BPM (one IEL) as our computational domain
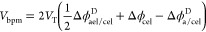
2where Δϕ_ael/cel_^D^ is the Donnan potential at the junction (i.e., AEL/CEL interface), Δϕ_cel_ the potential drop across the inner region of the CEL, and Δϕ_a/cel_^D^ the Donnan potential at the CEL/acid solution interface. The factor 2 is because our computational domain is half of a BPM, and so it must be multiplied by 2 for voltage across the entire membrane. Each term within the parentheses is dimensionless, and can be multiplied by the thermal voltage, *V*_T_ = *RT*/*F* (about 25.6 mV at room temperature), to return to a dimensional potential.

At all IEL-solution interfaces, we assume Donnan equilibrium for all ions, and thus the ion concentration just inside the IEL, *c*_*i*_, relates to that just outside, *c*_*i*,*∞*_, by the Boltzmann equation
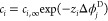
3where we neglect a nonelectrostatic contribution to the partitioning of the ions.^33^ We assume that concentrations of ions in solution right next to the membrane are the same as in bulk. Thus, also pH is the same right at the membrane as in bulk. The effect of concentration changes through the boundary films likely becomes important to consider at high current densities.

We model the BPM junction as an infinitely thin reservoir, and can thus apply the Boltzmann equation to the junction as well. This approach is similar to the virtual reservoir concept—a key element in space charge theory for nanofiltration.^34−36^ Ion concentrations in the junction are not known upfront but are predicted by the model, and they are highly sensitive to the magnitude and direction of the applied current: under forward bias, the salt concentration in the junction can be several moles per liter (M), while under reverse bias, the junction contains only deionized water with trace amounts of Na^+^ and Cl^–^.

At high current densities, ion concentration profiles across the BPM, including of H^+^ and OH^–^ ions, will be more extreme, leading to a more pronounced charge regulation effect, and the BPM may suffer membrane charge reduction (“charge regulation” or “current-induced membrane discharge”) because of a reaction between protons/hydroxyl ions and the fixed membrane charges.^37^ To account for this, we include in the model (in addition to diffusion and electromigration of all ions) that the membrane charge density, *ωX*, depends on the local pH by a protonation/deprotonation reaction. For instance, for the negatively charged CEL the membrane charge can be neutralized by proton adsorption. We describe this local protonation equilibrium by a Langmuir adsorption isotherm, which implies that the (position-dependent) CEL charge is described by
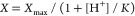
4The proton adsorption constant, *K*, with unit mM relates to the p*K* of proton adsorption by p*K* = 3 – log_10_*K*, while [H^+^] is the local proton concentration at that position in the CEL. Note that when we include these ionization reactions, the sum of the p*K* values for the AEL and CEL must be equal to 14 (e.g., if the p*K* of the CEL is set to 1.0, then the p*K* of the AEL is 13.0) to retain the validity of the symmetry assumption for the entire BPM calculation.

The aforementioned equations are discretized along the *x*-coordinate, and the resulting set of coupled nonlinear algebraic equations are solved numerically for steady state across a BPM, as explained in refs (31, 38, 39). Interestingly, unlike other BPM modeling approaches,^40−45^ we do not include any kinetic rate of the water recombination or dissociation in the BPM junction in this modeling framework. Instead, we reckon these reactions are fast enough to have chemical equilibrium between H^+^ and OH^–^ at all times. Making this assumption allows us to simplify the model significantly, as we can exclude incorporating any kinetic rates into the model while taking into account, albeit indirectly, the effect of the BPM catalyst—we assume that the catalyst that is in the junction between the two ion exchange layers successfully enhances the water splitting reactions, such that these reactions are no longer rate-limiting. Therefore, in the model, we only have to describe transport of ions inside the ion exchange layers.

### Analytical Solution for Limiting Current Density

A typical *I*–*V* plot of BPMs in neutral pH salt solution includes a current plateau, commonly named limiting current region (Figure 1b). However, the limiting current effect in BPMs is different from the standard limiting behavior observed in the electrodialysis process where it relates to the concentration polarization at the membrane/solution interface due to transport-limited processes.^46−50^ In BPMs, the limiting current is the result of ion depletion inside the BPM’s ion exchange layers.^51,52^ It corresponds to a condition at which water dissociation does not dominate yet and the current is carried mainly by salt ions. Thus, we want to emphasize that although we will refer to the plateau region as the limiting current region throughout this paper, the origin of the “limiting current” is different for “monopolar” and for bipolar membranes.

We can derive a (semi)analytical model for the BPM under forward bias that is valid for conditions where the current is carried mainly by salt ions. That is until, and including, the limiting current density which corresponds to the plateau region of the polarization curve. Thus, for the analytical solution, we do not need to consider any water ions (H^+^ and OH^–^) in the membrane and can restrict the calculation to Na^+^ and Cl^–^ only.

Because of the symmetry assumptions, the fluxes of Na^+^ and of Cl^–^ are equal and opposite to each other, i.e., . We can then derive for the current density
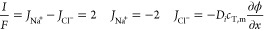
5with *D*_*i*_ the ion diffusion coefficient, that we assumed to be the same for anions and cations and with the total ion concentration . We can combine eq 5 with the Nernst–Planck equation for either Na^+^ or Cl^–^ in either AEL or CEL, and jointly with an expression for local electroneutrality we arrive at
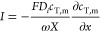
6which can be integrated to
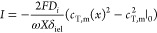
7resulting in the concentration profiles of the salt ions in each layer as a function of position *x* (for instance for the counterion, ). Molar fluxes of each ion are invariant across the entire BPM and equal in value but opposite in direction.

In eq 7, position 0 is for instance *x* = 0 on the left side of the AEL, i.e., next to the external solution with salt concentration, *c*_*∞*_. We can then solve eq 7 for *x* = δ_iel_ at the interface of the AEL and the junction with an unknown salt concentration. This salt concentration in the junction (which follows from the calculation) is higher than *c*_*∞*_ for forward bias, while for reverse bias it is lower, with values much less than 1 mM in the region where current levels off (limiting current). We can insert concentration profiles predicted by eq 7 in the Nernst–Planck equation for the flux of either of the ions, integrate again and arrive at an expression for the potential drop across each IEL, Δϕ_iep_. Using an analytical approach, we can calculate the four Donnan potentials for each value of *c*_*∞*_ in solution and in the junction, and with eq 7 we can calculate the current and Δϕ_iep_. Finally, we can calculate the total BPM voltage *V*_bpm_, thus constructing the full polarization curve.

According to this analytical model, at sufficiently high current densities under reverse bias operation the salt concentration in the junction goes to zero. This implies that the concentration of counterions in the membrane just next to the junction approaches the value of the fixed membrane charge, and thus the limiting current is given by
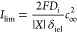
8which predicts the limiting current to scale with external salt concentration to the power 2.^53^

## Experimental Section

### Experimental Setup

The experiments were performed in an electrochemical flow cell made of poly(methyl methacrylate) (PMMA). Four ion exchange membranes (Fumasep FAB/FKB/FBM, Fumatech BWT GmbH, Germany), each with an active area of 5.3 × 5.3 cm^2^, were arrayed (AEM-CEM-BPM-AEM) to separate the cell into five compartments, each with a working volume of 56 mL. The three central compartments were for salt, base, and acid solutions. The two external compartments, both housing a 5.3 × 5.3 cm^2^ platinum–iridium coated (50 g/m^2^) titanium electrode (MAGNETO Special Anodes B.V., The Netherlands), were for anolyte and catholyte. A feed solution of 500 mL for each compartment was recirculated with a peristaltic pump (Masterflex L/S, Cole-Parmer, USA) at a flow rate of 170 mL/min. Various feed conditions were tested in the central compartments, using either NaCl or HCl and NaOH at different concentration in Milli-Q water. The anolyte and catholyte were always acidified aqueous solution of 0.25 M FeCl_2_, 0.25 M FeCl_3_. Two Ag/AgCl reference electrodes (QM711X, QIS, The Netherlands) were used to measure the voltage over the BPM. The reference electrodes were connected through a salt bridge to two Luggin capillaries, one on each side of the BPM, for a close and precise measuring point. During experiments with only salt solution in the central compartments, 3 M KCl was used as the salt bridge solution. In other instances, HCl or NaOH solution at the same concentration as the feed was used as bridge solution in acid and base compartments, respectively. All the electrochemical measurements were performed at a four-electrode configuration using a potentiostat (Iviumstat, Ivium Technologies, The Netherlands).

### Experimental Procedure

All membrane samples were preconditioned by immersing them in NaCl solution at the testing condition concentration for at least 24 h. Before measurements with acid and base solutions, an additional “desalting” pretreatment step was added. During the desalting step, the BPM was assembled into the cell with CEL facing the cathode (i.e., under reverse bias) and with acid and base as feed solutions. Next, a small current density (4 mA/cm^2^) was applied for ∼4 h to “draw out” the salt ions from the membrane. After finishing the pretreatment steps, all solutions were freshly made and changed for testing. During current–voltage measurements, voltage was applied over the BPM in increments of 20 mV from −0.1 to 1 V for tests with salt solutions, and from 0.5 to 1 V for tests with acid and base solutions. Each voltage step was applied for 20 s, and the resulting current was recorded at the end of each step.

## Results and Discussion

All the modeling results in this section had the following input parameters: membrane thickness δ of 200 μm, membrane fixed charge density *X* of 5 M, salt ion diffusion coefficients in the membrane  and  of 4.0 × 10^–11^ m^2^/s (50 times smaller than in bulk solution), and water ion diffusion coefficients  and  five times that of salt, 2.0 × 10^–10^ m^2^/s.

### BPM Polarization Curve in Neutral Solutions

**Figure 2:**
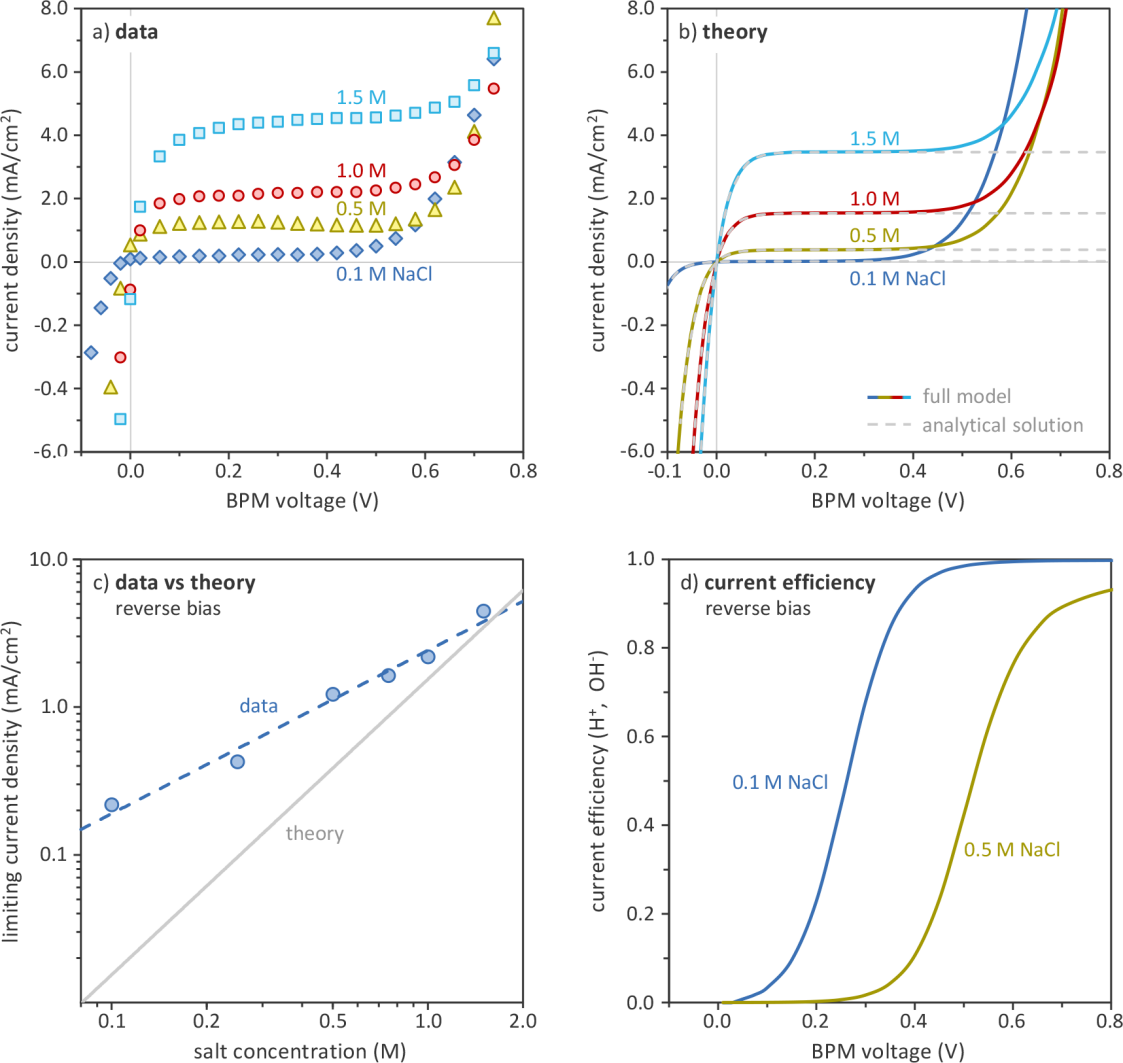
Effect of NaCl bulk concentration on BPM current–voltage curve: (a) experimental data; (b) model predictions; (c) relation between limiting current density and NaCl bulk concentration, according to experimental data and theory; (d) theoretical current efficiency of water dissociation (OH^–^ and H^+^) as a function of BPM voltage.

A commercial BPM (Fumasep FBM), tested with NaCl solution on both sides, exhibits a typical current–voltage behavior for a BPM in neutral pH salt solution (Figure 2a).

Our theory can reproduce the full *I*–*V* curves, as demonstrated in Figure 2b. Under forward bias (negative current values) the current first changes linearly but beyond a certain voltage increases steeply. Under forward bias current is carried by Cl^–^ and Na^+^ ions only, as they are driven from the external solution into the junction. Once the concentration of salt in the junction equals the fixed charge concentration of the BPM (see Figure 5 for more details), the selectivity of each layer drops significantly, and Cl^–^ and Na^+^ cross the CEL and AEL, respectively, as co-ions. Thus, high currents can be achieved under forward bias when applying a voltage high enough to overcome Donnan exclusion. However, at such conditions, the BPM may suffer from delamination due to excessive salt precipitation or water formation (from osmotic pressure due to high salt concentration at the junction, or from OH^–^ and H^+^ recombination when operating the BPM in acid and base) at its junction.^11,26^

Under reverse bias (positive current values), there is a narrow linear region at low currents, followed by a limiting current plateau, and then an overlimiting current region where current increases rapidly. In the first two regions, the current is mainly carried by salt ions. In the overlimiting region, however, the importance of water dissociation—which creates H^+^ and OH^–^ ions—dominates (and thus the assumption in the model that we always have pH 7 on the outsides of the membrane may not be accurate at high current density). Contrary to early literature on BPMs, which hypothesized that water dissociation starts only at a certain high enough “offset” voltage, more recent works have demonstrated that water ions carry a significant portion of current even at current densities below 1 mA/cm^2^.^54,55^ This leads to a premise that the dominance of water dissociation develops gradually, which our calculations for current efficiency of water dissociation confirm (Figure 2d).

Yet the predicted (and experimental) *I*–*V* curves still exhibit a distinct change of slope that marks the transition from limiting current region to overlimiting current region, indicating that the contribution of water ions to the current starts to dictate the membrane’s current–voltage behavior from some critical point onward (corresponding to the “start” of the overlimiting region). One explanation for the rapid increase of current beyond a certain voltage is that the kinetics of water dissociation are enhanced once the electric field at the BPM junction becomes sufficiently high (Second Wien effect).^43,44,56,57^ However, our model that we used to obtain Figure 2 panels b and d does not include any description of the kinetic rate of water dissociation. Instead, we assume that the kinetics of the water dissociation reaction are infinitely fast. Our results indicate that we do not need to include a possible role of the electric field on the rate of water dissociation to simulate current–voltage curves that exhibit a clear overlimiting region with a current “takeoff”.

An effect of the bulk electrolyte concentration can be observed on the limiting current density value, as well as on the plateau “length”, both in our theory and in our experimental results (Figure 2a, b). Note that our theory does not include boundary layer effects that may play a role outside of the membrane, as discussed in ref (58).

In an ideal BPM, no ions should be transported across both layers of the membrane. Therefore, the limiting current plateau could be seen as a measure of the BPM selectivity. According to the results shown in Figure 2a, b, limiting current density increases with bulk electrolyte concentration, indicating a decrease of the overall BPM selectivity. As a result, a larger fraction of current is carried by salt ions and thus the overlimiting region (i.e., where current starts to increase rapidly) shifts to higher voltages. Salt ion crossover lowers water dissociation efficiency because the charge otherwise used to dissociate water and release OH^–^ and H^+^ ions from the junction is carried by salt ions instead. This effect is illustrated well in Figure 2d, which shows the current efficiency (based on OH^–^ and H^+^) as a function of the voltage over the membrane.

**Figure 3:**
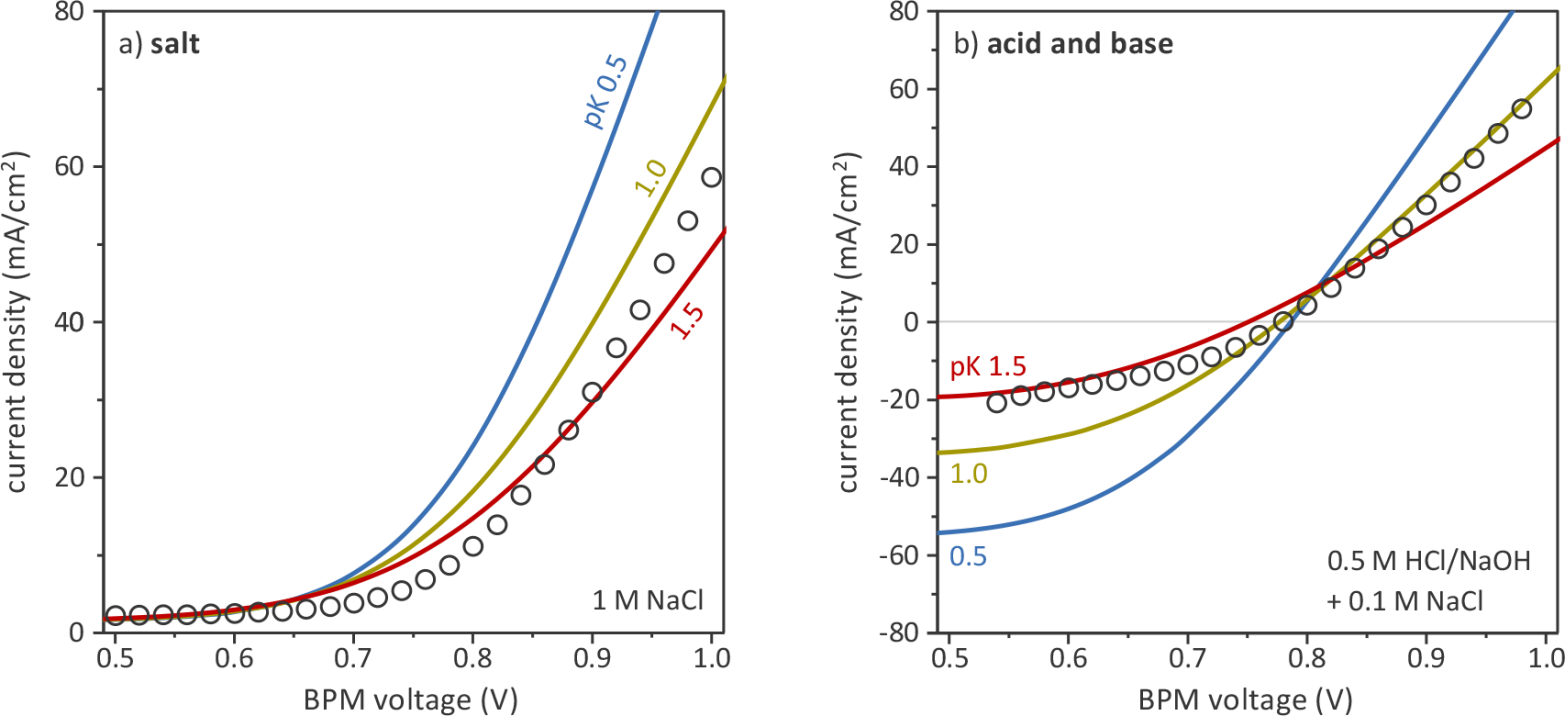
Comparison between model (lines) and experimental data (symbols) at different CEL p*K* values. (a) Water dissociation in 1 M NaCl; (b) water dissociation/formation in 0.5 M HCl/NaOH with 0.1 M NaCl.

The developed analytical model (Figure 2b) predicts well all the *I*–*V* curve regions where salt ions carry majority of the current: the forward bias region and the limiting current region, but not the overlimiting region where current is mainly carried by the H^+^ and OH^–^ ions. The effect of *c*_*∞*_ on the *I*_lim_, however, is overestimated (Figure 2c): the experimental data show a direct proportionality between *I*_lim_ and *c*_*∞*_ instead of a quadratic dependence predicted by the theory (eq 8). The experimental limiting current densities are 0.24, 1.2, 2.2, and 4.4 mA/cm^2^ and theoretical values 0.02, 0.38, 1.55, and 3.45 mA/cm^2^ for NaCl concentration of 0.1, 0.5, 1.0, and 1.5 M, respectively. To see the effect of membrane charge reduction on the *I* – *V* curve, we then tried using a fixed membrane charge in the model instead. We have not presented these results in Figure 2 because interestingly, only the overlimiting region of the *I*–*V* curve is slightly modified, with the slope somewhat steeper when we assume a fixed charge, but the theoretical prediction of power 2 dependence of *I*_lim_ on *c*_*∞*_ remained unchanged. We have no explanation for this discrepancy between the data and theory (Figure 2c). Because the experimentally measured limiting current densities are consistently higher than those predicted by the model, and because in the limiting current region the current is carried to a large extent by salt ions, one possible explanation is that the membrane is less selective than assumed in the model. Another discrepancy between data and theory we must acknowledge is in the overlimiting region. The model includes only the membrane in its computational domain, and no external solution. Because the resistance of a material (here the membrane) is constant, the predicted current–voltage slopes in the overlimiting region result parallel. In practical measurements, however, the external solution and the associated boundary layers will affect the measurements, and especially at low bulk concentrations. To replicate the experimental data at low salt concentration with theoretical calculations better, we introduced the effect of the external solution resistance (see Figure S1 in the Supporting Information). Nevertheless, other aspects of the *I*–*V* curve for BPMs are very well reproduced, with p*K* value between 1 and 1.5 for CEL (and thus 13–12.5 for AEL), giving the best match with the experimental data, as demonstrated in Figure 3. p*K* and local pH have an effect on the ionization of the charged groups, and thus they affect the membrane charge. The performance of the membrane improves significantly with decreasing p*K* for CEL.

### Effect of Background NaCl Electrolyte in Acid–Base Solutions on BPM Polarization curve

**Figure 4:**
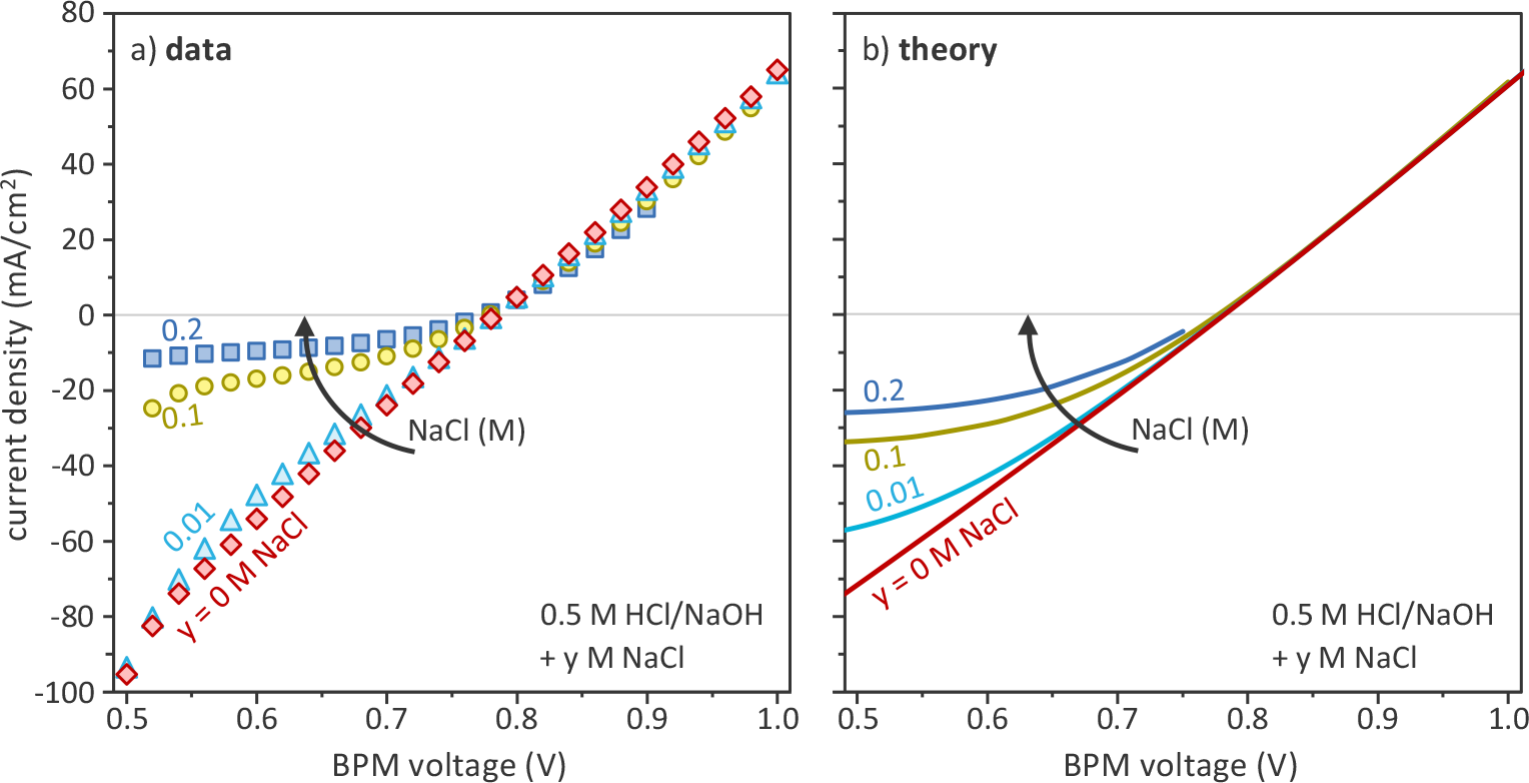
Effect of NaCl concentration on BPM current–voltage curve in acid–base conditions (0.5 M HCl/NaOH). (a) Experimental data; (b) model predictions.

Bipolar membranes are typically tested under steady-state polarization sweep using pure electrolyte (e.g., NaCl) solutions.^1^ However, in practical applications where acid/base and other electrolytes are present in the bulk as a multi-ionic mixture, the performance of BPM will be affected by the transport of all co-ions. To experimentally investigate such conditions, we have explored the BPM current–voltage behavior at extreme pH conditions by using 0.5 M HCl (CEL side) and NaOH (AEL side) solutions with increasing concentration of background NaCl electrolyte as feed (Figure 4). Under such conditions, several differences can be observed on the BPM *I*–*V* curve: for instance, a nonzero open circuit voltage (OCV) can be measured, according to the emerging Donnan potential difference at both sides of the BPM. Moreover, no visible limiting current region can be observed. Although the salt ions carry a similar portion of the overall current independent of the pH conditions, at extreme pH conditions the contribution of salt ions remains obscured as a result of the high OH^–^/H^+^ concentration in the bulk.^44^ In the case of pure acid and base feed (i.e., no additional NaCl as background electrolyte), the current is carried mainly by H^+^ and OH^–^ ions throughout the entire *I*–*V* curve, and the curve is symmetric (in forward and reverse bias) with respect to the OCV. This result has been previously observed both experimentally^5^ and theoretically.^44^

The effect of increasing NaCl concentration has a noticeable impact on the resulting *I*–*V* curve. In particular, under reverse bias (i.e., where the membrane is in the water dissociation mode) Cl^–^ and Na^+^ do not significantly affect the membrane performance, because they can only enter the membrane as co-ions. We did not observe a remarkable change in the current–voltage behavior neither experimentally nor theoretically in this case. However, the situation is different when the BPM is under forward bias (water formation), where the effect of background NaCl is significant. Under this condition, all ions are driven by the external electric field from the outer solutions into the membrane (i.e., toward the junction), and thus the Cl^–^ (or Na^+^) ions in the bulk solution are competing with OH^–^ (or H^+^) ions as counterions. This results in a dramatic reduction of the current density at any given voltage as observed both experimentally and theoretically (Figure 4). The higher the concentration of the background salt, the higher the ratio of current carried by less mobile salt ions instead of water ions. Thus, compared to the case of pure acid and base, higher voltages are needed to reach similar current densities. Such strong asymmetric effect of NaCl on the BPM’s electrical behavior under forward vs reverse bias is crucial in applications that involve both biases (e.g., acid–base flow batteries), as it results in efficiency loss during cycling. During the battery charging step, salt solution is converted into acid and base. However, due to nonideal selectivity of the BPM (as well as monopolar membranes), some salt will remain in the acid and base solutions. During the battery discharge (water formation), the presence of background electrolyte in high concentration will result in a voltage loss, thus lowering the round-trip efficiency. Particularly, the larger the salt ion flux, the more asymmetrical the BPM behavior, which is detrimental for battery applications. The situation is different for BPM-assisted fuel cells, which operate consistently under forward bias using only H_2_ and O_2_ gases as feed,^12^ and can thus ideally avoid introducing any salt ions into the system.

**Figure 5:**
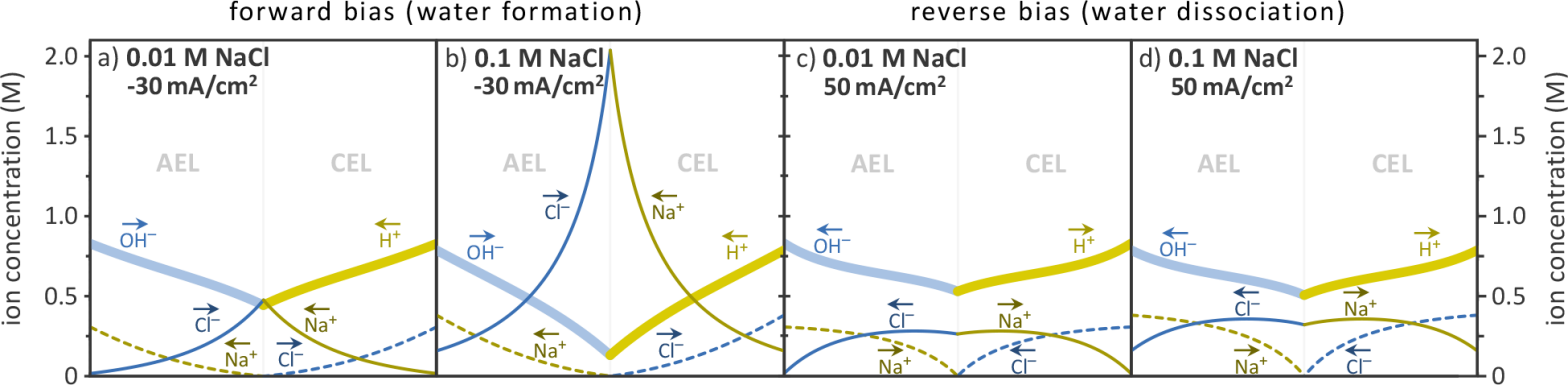
Ion concentration profiles inside AEL and CEL in 0.5 M HCl/NaOH solutions at different background NaCl concentration (0.1 and 0.01 M NaCl). (a, b) Forward bias (−30 mA/cm^2^). (c, d) Reverse bias (+50 mA/cm^2^). Arrows in the figure indicate the direction of ion transport.

To further explain theoretically the impact of background salt on the BPM behavior, we calculated the concentration profiles of all four ions (Na^+^, Cl^–^, H^+^, OH^–^) inside the AEL and CEL for both operational modes (forward/reverse bias) as presented in Figure 5. We considered a case where the BPM separates a 0.5 M HCl solution (CEL side) and a 0.5 M NaOH solution (AEL side), with either 0.01 or 0.1 M NaCl background electrolyte in both acid and base. Since the calculation is based on the assumption of perfect symmetry, the concentration profiles of Na^+^, Cl^–^ and H^+^, OH^–^ are exact mirror images of one another as shown in Figure 5. Equation 7 indicates that as long as H^+^ and OH^–^ do not participate in current transfer (mainly before the overlimiting current region), concentrations of co-ions and counterions have a parabolic profile. In Figure 5, that is no longer the case, as here we have chosen conditions where H^+^ and OH^–^ carry a significant current.

Under forward bias (Figure 5a, b), ions migrate toward the junction: OH^–^ and Cl^–^ through AEL, and H^+^ and Na^+^ through CEL. OH^–^ and H^+^ ions recombine in the junction and form water which flows out of the membrane. The salt ions, however, can exit the membrane only as co-ions and their concentration in the junction goes to a high value. Based on our predictions, 0.01 or 0.1 M NaCl concentration in bulk solution results in 50× or 20× higher salt concentration in the BPM junction, accordingly. It is noteworthy that for the 0.1 M background salt, this corresponds to around 2 M NaCl concentration at the junction. At such high salt concentration the membrane selectivity drops significantly, and as a result more Cl^–^ and Na^+^ ions are transported through the CEL and AEL, accordingly, as co-ions. The effect of NaCl under reverse bias is less significant (i.e., with smaller differences between panels c and d in Figure 5). Here the salt ions can enter the membrane only as co-ions. Once a co-ion reaches the junction, it can easily exit the membrane from the other layer as a counterion.

## Environmental Implications

The Nernst–Planck modeling framework presented in this work is a new approach for water dissociation at the BPM junction. With respect to previous works, our model is simple, requiring only a few membrane parameters (i.e., thickness, charge density, and p*K* of proton adsorption) to fully predict current–voltage curves of BPMs. Although the model overestimates the dependence of limiting current on salt concentration, it can still predict qualitatively well all the different regions of a commercial BPM’s current–voltage curve (forward bias, reverse bias including limiting current region and overlimiting current region) and simulate theoretical concentration profiles and fluxes of ions (in this work H^+^, OH^–^, and Na^+^, Cl^–^) inside the membrane, at both neutral and extreme pH conditions.

Nonetheless, several improvements may still be made to the model to make it more mechanistically accurate, which may be required depending on the intended application. For example, the present theory does not include the relevance of bulk water transport. Accordingly, our theory cannot predict the second limiting current region where water transport from bulk solution into the membrane becomes limiting. Such water transport limiting behavior, however, is typically encountered at very high current densities (e.g., 500 mA cm^2^).^59^ Theoretically, water has multiple transport pathways—flow through pressure gradients and transport via migration of hydrated ions. Thus, the ions in the membrane are convected with the water which modifies their transport rates. Additionally, the diffusion coefficients of Na^+^ and Cl^–^ can be different in AEL and CEL. For instance, Na^+^ being transported as a counterion in the CEL may have a lower diffusion coefficient than when it is a co-ion in the AEL.^38^ Furthermore, we assume that the membrane charge is influenced by local pH, i.e., due to protonation or deprotonation of membrane charges, but it may be also possible that the membrane charge is effectively reduced because of Na^+^ or Cl^–^ ions’ adsorption into the membrane.^60^ We have also not included current induced phenomena, such as pressure^61^ and temperature^62^ changes, which may also affect ion and water transport rates. Water dissociation and formation, too, are accompanied by thermal effects. Lastly, we have not considered possible pH changes through the external boundary layers. Though changes in salt concentration are likely small in these layers because currents are not very high, the fluxes of H^+^ and OH^–^ entering and leaving the membrane will have a strong effect on the local pH near the membrane, especially in the case with neutral pH salt solution.

We emphasize that although the model can be expanded on to include the described phenomenon, good agreement between our experimental and model results indicates that the essence of water dissociation in BPMs is captured by the current framework. Thus, the presented theory predicts to a good degree the limiting current density and current response of BPMs under operation in various salt or acid/base solutions, which may be sufficient for initial stages of technology development and evaluation.

Regarding the water dissociation theory presented, it is notable that we did not incorporate kinetic rates of water dissociation and recombination, unlike other modeling frameworks in the literature. Even without the specification of the kinetics, the model can still predict a BPM’s full current–voltage curve. Our results thus indicate that the role of the electric field on the rate of water dissociation (Second Wien effect) may have been overstressed in prior works. The good agreement between our model and experimental results using commercial membranes suggests that it is sufficient to assume water dissociation and recombination reactions are fast enough to have chemical equilibrium between H^+^ and OH^–^ at all times. Thereby, our results also indirectly indicate that the water dissociation catalyst used in the tested commercial membrane works sufficiently well. Development of catalysts that further accelerate the rate of water dissociation—beyond the rates attainable in commercially available BPMs—may thus be of little practical advantage.

Our results, which considered the often overlooked forward bias regime, also provide new insight into the asymmetric current–voltage behavior of the BPM and demonstrate how salt ions affect the membrane performance under forward bias significantly more than under reverse bias. This strong asymmetric effect of salt ions helps explain why the BPM applications involving alternating forward–reverse bias operational modes (e.g., acid–base flow batteries) are especially sensitive to salt ion crossover, and suffer from poor round-trip efficiency in case of high background salt electrolyte concentration in acid/base solutions. For such applications, novel process-tailored BPMs need to be developed (i.e., membranes with specific selectivity toward H^+^ and OH^–^) to reduce the effect of co-ion transport and to withstand high current densities under forward bias (water formation) conditions.

As the number of technologies that use BPMs continues to grow, it is increasingly important to investigate the performance of BPMs beyond the standardized conditions of pure NaCl solutions for acid–base production. In this work, we demonstrated BPM behavior under both reverse (water dissociation) and forward (water formation) bias at different operating environments, with Figure 3 and 4 covering most of the practical operating conditions for current BPM applications. Nonetheless, we note that our modeling framework may be readily altered and expanded on for modeling BPM behavior in various electrolytes. Such capability is of foreseeable importance as emerging BPM applications have been applied to a wide variety of source water compositions and salinities. For example, a BPM-integrated electrosorption process was suggested for boron removal from low salinity first pass seawater reverse osmosis permeate,^25^ while on the other end of the spectrum, bipolar membrane electrodialysis has been applied for valorization of hypersaline reverse osmosis brines.^63^ Future iterations of the presented framework should thus focus on ensuring the accuracy of BPM performance over a wide salinity range, with particular emphasis on improving prediction capability in the low salinity regime, where the external solution and the associated boundary layers become important to consider, and where our current model shows the largest discrepancy from experimental data. Bipolar membranes are also used in CO_2_ electrolyzers,^64^ lithium recovery,^3^ ammonia recovery,^8^ carbon capture,^6^ and heavy metals removal^65^—all of which introduce greater system complexity due to the necessary consideration of multiple ionic species. Hence, the use and extension of a simplistic, yet accurate BPM modeling framework, as presented in this work, is advantageous for environmental process development and optimization. Furthermore, as illustrated in this study, the mechanistic nature of the developed model makes it a useful tool for highlighting process bottlenecks (e.g., we revealed that substantial crossover of salt ions in acid–base flow batteries limits round-trip efficiency). Such mechanistic insight will be required for assessing the potential and practical limitations of emerging BPM technologies.

## Supporting Information Available

Effect of bulk electrolyte on current–voltage measurements; model predictions corrected for bulk electrolyte *iR*-drop (PDF)
