# Supplementary material for: Origin of Limiting and Overlimiting Currents in Bipolar Membranes
Source: Environ Sci Technol. 2023 Jun 21;57(26):9664–74. doi: 10.1021/acs.est.2c09410 (PMC10324307; doi:10.1021/acs.est.2c09410)
Supplement: Supplementary file 1 — es2c09410_si_001.pdf [file es2c09410_si_001.pdf]

Supporting Information for

The origin of limiting and overlimiting currents in bipolar membranes

Ragne Pärnamäe*, Michele Tedesco, Min-Chen Wu, Chia-Hung Hou, Hubertus V.M. Hamelers, Sohum K. Patel, Menachem Elimelech, P.M. Biesheuvel, and Slawomir Porada*

* Corresponding author. Email: slawomir.porada@pwr.edu.pl (S.P.); ragne.parnamae@wetsus.nl (R.P.)

This PDF file includes:

Supporting text

Figure S1

Supporting text

Effect of electrolyte bulk concentration on current-voltage measurements.

The use of Luggin capillaries allows moving the reference electrode's sensing point close to the BPM-solution interface. However, a small gap between the capillary tip and the BPM needs to remain to avoid current shielding, implying that there exists a bulk electrolyte solution layer between the capillary tip and the BPM surface, which is included in the measurement. With decreasing values of electrolyte concentration, the resistance in bulk increases, and from a particular concentration the resistance of the electrolyte solution layers will dominate the total resistance. This leads to a significant error in the measured BPM voltage drop.

Supporting figures

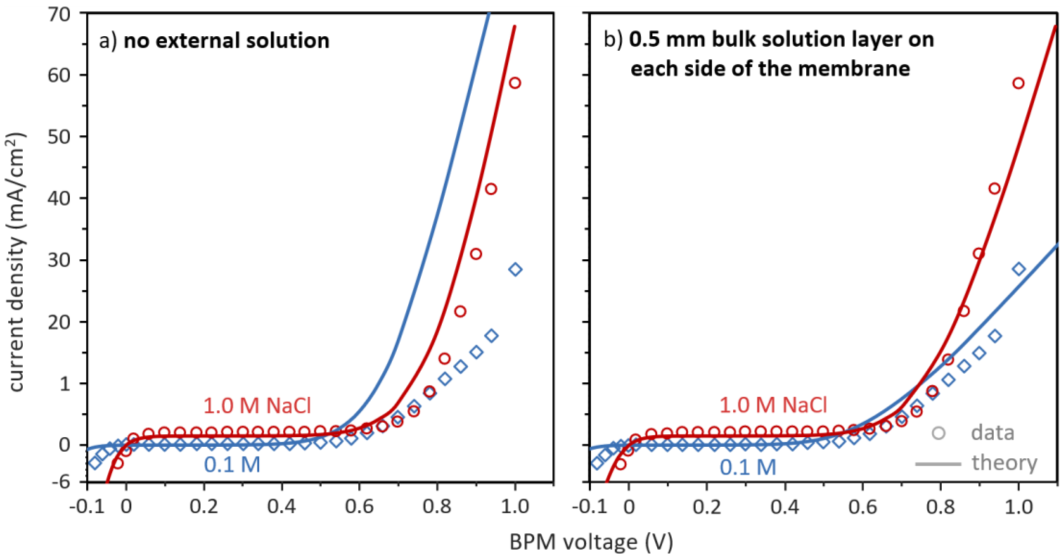

Figure S1: The effect of electrolyte solution resistance on BPM current-voltage curve. a) experimental data and model predictions as presented in Fig.2a-b in the manuscript body, with extended axes; b) the same experimental data but model predictions have been corrected for bulk-iR-drop, assuming there is a 0.5 mm layer of NaCl solution at the tested concentration (conductivity 1.0 S/m for 0.1 M and 7.1 S/m for 1 M NaCl solution) on each side of the membrane.
